# Formation of virus-like particles from human cell lines exclusively expressing Influenza Neuraminidase

**Published:** 2011-01-10

**Authors:** JCC Lai, WWL Chan, JM Nicholls, JSM Peiris, JM Garcia

**Affiliations:** 1 HKU-Pasteur Research Centre, Hong Kong, Hong Kong SAR; 2 Department of Pathology, The University of Hong Kong, Hong Kong SAR; 3 Department of Microbiology, The University of Hong Kong, Hong Kong SAR

## Background

Neuraminidase (NA) is an important viral component of influenza viruses and the target of most effective anti-influenza drugs in the market. Most of the data on NA function obtained so far has come from using native virions (which imposes bio-safety issues) or from purified NA proteins which may not have the same properties as that on viral surfaces [1]. We have developed virus-like particles (VLPs) containing only the NA, functionally and morphologically similar to the native virions. NA-VLPs may be useful in influenza research such as the investigation of the assembly and budding steps in the virus life cycle as this process is still unclear [2-5].

## Methods

In order to determine the minimal set of viral proteins essential for virus budding, 293T cells were transfected with plasmids encoding for the haemagglutinin (HA), NA and matrix (M1) proteins singly, or in combination. VLP released into the culture medium were collected by ultra-centrifugation and their protein composition analyzed by western blotting. Budding of the VLPs were visualized by electron microscopy. Kinetics of the production of VLP containing solely NA was monitored by enzymatic activity assays and western blotting. Physical and functional characterization of the NA-VLP were carried out using (i) sucrose gradient centrifugation, (ii) neuraminidase activity assay, (iii) NA oligomerization analysis, as well as (iv) lectin staining of sialic acid on cell surface. In addition, the effect of NA enzymatic activity on VLP production was investigated by using sialidase inhibitor or a point mutation (E262D) in NA that inactivates the catalytic site.

## Results

VLP formation was detected from cells expressing HA and NA alone but not from cells solely expressing M1 showing that HA an NA each contributes to the driving force for virus budding whereas M1 only had a limited contribution. The sialidase inhibitor and E262D point mutation studies showed that the enzymatic activity of NA is not required in driving virus budding of NA VLPs. However, release of VLP containing HA was completely dependent on sialidase, either as co-expressed NA or added exogenously. NA-VLPs were morphologically similar to influenza virions by electron microscopy and NA on the VLP surface was chemically and functionally comparable to that on infectious virus particles.

## Conclusion

NA plays a key role in virus budding and morphogenesis at least for the N1 subtype that we studied. These NA-VLPs mimic NA from native virions and represents a very useful tool in influenza research [6].
